# A Genetics-First Approach Revealed Monogenic Disorders in Patients With ARM and VACTERL Anomalies

**DOI:** 10.3389/fped.2020.00310

**Published:** 2020-06-23

**Authors:** Romy van de Putte, Gabriel C. Dworschak, Erwin Brosens, Heiko M. Reutter, Carlo L. M. Marcelis, Rocio Acuna-Hidalgo, Nehir E. Kurtas, Marloes Steehouwer, Sally L. Dunwoodie, Eberhard Schmiedeke, Stefanie Märzheuser, Nicole Schwarzer, Alice S. Brooks, Annelies de Klein, Cornelius E. J. Sloots, Dick Tibboel, Giulia Brisighelli, Anna Morandi, Maria F. Bedeschi, Michael D. Bates, Marc A. Levitt, Alberto Peña, Ivo de Blaauw, Nel Roeleveld, Han G. Brunner, Iris A. L. M. van Rooij, Alexander Hoischen

**Affiliations:** ^1^Department for Health Evidence, Radboud Institute for Health Sciences, Radboud University Medical Center, Nijmegen, Netherlands; ^2^Department of Pediatrics, Children's Hospital, University Hospital Bonn, Bonn, Germany; ^3^Institute of Human Genetics, University of Bonn, Bonn, Germany; ^4^Department of Clinical Genetics, Erasmus Medical Centre, Rotterdam, Netherlands; ^5^Department of Pediatric Surgery, Erasmus Medical Centre—Sophia Children's Hospital, Rotterdam, Netherlands; ^6^Department of Neonatology, Children's Hospital, University Hospital Bonn, Bonn, Germany; ^7^Department of Human Genetics, Radboud Institute for Molecular Life Sciences, Radboud University Medical Center, Nijmegen, Netherlands; ^8^Victor Chang Cardiac Research Institute, UNSW Sydney, Sydney, NSW, Australia; ^9^Department of Pediatric Surgery and Urology, Centre for Child and Youth Health, Klinikum Bremen-Mitte, Bremen, Germany; ^10^Department of Pediatric Surgery, Campus Virchow Clinic, Charité University Hospital Berlin, Berlin, Germany; ^11^SoMA e.V., Self-Help Organization for People With Anorectal Malformation, Munich, Germany; ^12^Department of Paediatric Surgery, Chris Hani Baragwanath Academic Hospital, Johannesburg, South Africa; ^13^Department of Pediatric Surgery, Fondazione IRCCS Ca' Granda—Ospedale Maggiore Policlinico, Milan, Italy; ^14^Medical Genetic Unit, Fondazione IRCCS Ca' Granda—Ospedale Maggiore Policlinico, Milan, Italy; ^15^Division of Gastroenterology and Nutrition, Dayton Children's Hospital, Dayton, OH, United States; ^16^Department of Pediatrics, Boonshoft School of Medicine, Wright State University, Dayton, OH, United States; ^17^Department of Surgery, Center for Colorectal and Pelvic Reconstruction, Nationwide Children's Hospital, The Ohio State University, Columbus, OH, United States; ^18^Department of Surgery, International Center for Colorectal Care, Children's Hospital Colorado, University of Colorado, Aurora, CO, United States; ^19^Department of Surgery—Pediatric Surgery, Radboudumc Amalia Children's Hospital, Radboud University Medical Center, Nijmegen, Netherlands; ^20^Department of Human Genetics, Donders Institute for Brain, Cognition and Behaviour, Radboud University Medical Center, Nijmegen, Netherlands; ^21^Department of Clinical Genetics and School for Oncology & Developmental Biology (GROW), Maastricht University Medical Center, Maastricht, Netherlands; ^22^Department of Internal Medicine and Radboud Center for Infectious Diseases, Radboud University Medical Center, Nijmegen, Netherlands

**Keywords:** anorectal malformations, duane-radial ray syndrome, esophageal atresia, genetics-first, molecular inversion probe, Opitz-G/BBB syndrome, townes-brocks syndrome

## Abstract

**Background:** The VATER/VACTERL association (VACTERL) is defined as the non-random occurrence of the following congenital anomalies: Vertebral, Anal, Cardiac, Tracheal-Esophageal, Renal, and Limb anomalies. As no unequivocal candidate gene has been identified yet, patients are diagnosed phenotypically. The aims of this study were to identify patients with monogenic disorders using a genetics-first approach, and to study whether variants in candidate genes are involved in the etiology of VACTERL or the individual features of VACTERL: Anorectal malformation (ARM) or esophageal atresia with or without trachea-esophageal fistula (EA/TEF).

**Methods:** Using molecular inversion probes, a candidate gene panel of 56 genes was sequenced in three patient groups: VACTERL (*n* = 211), ARM (*n* = 204), and EA/TEF (*n* = 95). Loss-of-function (LoF) and additional likely pathogenic missense variants, were prioritized and validated using Sanger sequencing. Validated variants were tested for segregation and patients were clinically re-evaluated.

**Results:** In 7 out of the 510 patients (1.4%), pathogenic or likely pathogenic variants were identified in *SALL1, SALL4*, and *MID1*, genes that are associated with Townes-Brocks, Duane-radial-ray, and Opitz-G/BBB syndrome. These syndromes always include ARM or EA/TEF, in combination with at least two other VACTERL features. We did not identify LoF variants in the remaining candidate genes.

**Conclusions:** None of the other candidate genes were identified as novel unequivocal disease genes for VACTERL. However, a genetics-first approach allowed refinement of the clinical diagnosis in seven patients, in whom an alternative molecular-based diagnosis was found with important implications for the counseling of the families.

## Introduction

The VATER/VACTERL association (VACTERL) (OMIM %192350) is a very serious condition that is defined as the non-random combination of the following congenital anomalies: Vertebral, Anal, Cardiac, Tracheal-Esophageal, Renal, and Limb anomalies (1). The prevalence of VACTERL in Europe is 0.4 in 10,000, including live births, fetal deaths (miscarriages or stillbirths from 20 weeks of gestation), and termination of pregnancy following prenatal diagnosis of a fetal anomaly (2). Any combination of three features of VACTERL qualifies for a clinical diagnosis, resulting in large phenotypic heterogeneity among VACTERL patients (3–9). VACTERL usually occurs sporadically, but in some patients, familial occurrence of component features of VACTERL has been observed, indicating that genetic factors may play a role (10). However, other than variants identified in a few VACTERL patients, no candidate gene has been identified that explains the etiology in a substantial fraction of VACTERL patients yet (11–17). For example, biallelic mutations were recently identified in the 3-hydroxyanthranilic acid 3,4-dioxygenase (*HAAO*) gene and kynureninase (*KYNU*) gene in four unrelated patients with congenital vertebral, cardiac, and renal anomalies (17). Consequently, a VACTERL diagnosis is mainly based on the clinical phenotype.

Several monogenic disorders have phenotypic overlap with VACTERL, and patients should only get this diagnosis when the possibility of overlapping monogenic disorders is excluded (18). As monogenic disorders with phenotypic overlap are sometimes hard to distinguish based on clinical phenotype alone, a genetics-first approach may aid physicians in making more accurate molecular/genetic diagnoses. However, detailed genetic testing, e.g., whole exome sequencing (WES) or whole genome sequencing (WGS), is rarely performed in patients that exhibit congenital anomalies from the VATER/VACTERL malformation spectrum (1, 19).

In this study, we were interested whether a genetics-first approach for 26 genes would identify patients with monogenic disorders that were previously not diagnosed as such. In addition, we aimed to study the role of 30 candidate genes in the etiology of VACTERL or one of the component features of VACTERL: ARM or EA/TEF.

## Patients and Methods

### Study Population

We included three groups of patients diagnosed with: (1) VACTERL; (2) ARM with or without additional congenital anomalies; and (3) EA/TEF with or without additional congenital anomalies. Patients with ARM or EA/TEF with additional anomalies that do not fulfill the criteria for a diagnosis of VACTERL were included to study whether previously identified candidate genes for VACTERL are specific to VACTERL, or are also present in patients with the individual features ARM or EA/TEF. Patients were excluded when a genetically defined syndrome was diagnosed or highly suspected at the time of recruitment. The patients included had not been subjected to extensive genetic testing previously and were not necessarily seen by a clinical geneticist.

The patients were collected from different research groups: 84 VACTERL patients were derived from the German Network for Congenital Uro-REctal malformations (CURE-Net); 56 VACTERL patients from the Aetiologic research into Genetic and Occupational/environmental Risk factors for Anomalies in children (AGORA) data- and biobank (20) in Nijmegen, the Netherlands; 34 VACTERL patients from the Erasmus MC-Sophia Children's hospital in Rotterdam, the Netherlands; 29 VACTERL patients from Cincinnati Children's Hospital Medical Center, Cincinnati, Ohio, USA; and 8 VACTERL patients from Fondazione IRCCS Ca' Granda Ospedale Maggiore Policlinico in Milan, Italy. Furthermore, 201 ARM patients were included from CURE-Net, 3 ARM patients from Cincinnati Children's Hospital Medical Center, Cincinnati, Ohio, USA, and 95 patients with EA/TEF from the Erasmus MC-Sophia Children's hospital in Rotterdam.

The recently introduced VACTERL limits were used to classify our VACTERL cases in three mutually exclusive subtypes: STRICT-VACTERL, VACTERL-LIKE, and VACTERL-PLUS (9). The STRICT-VACTERL subtype contains cases with ≥3 major VACTERL features; the VACTERL-LIKE subtype contains cases with <3 major VACTERL features, but with additional minor VACTERL features adding up to ≥3 major and minor VACTERL features combined; and the VACTERL-PLUS subtype, contains cases that fulfilled either the STRICT-VACTERL or the VACTERL-LIKE subtype criteria, but had additional major congenital anomalies outside the VACTERL spectrum (9).

### Gene Panel Selection

The gene panel was selected based on three criteria: (1) genes known to cause human monogenic disorders that share features with VACTERL once mutated (*n* = 26); (2) candidate genes that were described previously in relation to VACTERL, ARM, or EA/TEF, either in animal models or in individual patients (*n* = 10) (12, 13, 16, 21–38); and (3) genes that cause congenital anomalies in three or more VACTERL organ systems in mouse knock-out models that resemble VACTERL features (*n* = 20). Some of the candidate genes listed in the first or second category also show mouse knock-out phenotypes as shown in Table 1. The final gene panel consisted of 56 genes.

**Table 1:** The candidate gene panel.

	**Gene**	**Genomic position (hg19)**	Mouse KO Phenotype*	**OMIM Disease association (inheritance)**
**Genes that result in human monogenetic disorders that share features with VACTERL**				
1	*AXIN1*	chr16:337439-402676	VRL	Caudal duplication anomaly (unknown)
2	*B9D1*	chr17:19240866-19281495	CRL	Meckel syndrome (AR), Joubert syndrome (AR)
3	*CC2D2A*	chr4:15471488-15603180	CRL	Joubert syndrome (AR), Meckel syndrome (AR)
4	*CHD7*	chr8:61591323-61780586		CHARGE syndrome (AD)
5	*DACT1*	chr14:59105554-59115038	VRL	Townes-Brocks syndrome (AD)
6	*DYNC2H1*	chr11:102980159-103350591	CTERL	Short-Rib polydactyly syndrome (AR, AD)
7	*EFTUD2*	chr17:42927654-42976993		Mandibularfacial dysostosis (AD)
8	*FANCA*	chr16:89803958-89883065		Fanconi anemia A (AR)
9	*FANCB*	chrX:14861528-14891184		Fanconi anemia B (XLR)
10	*FANCC*	chr9:97861335-98079991		Fanconi anemia C (AR)
11	*FANCD2*	chr3:10068112-10143614		Fanconi anemia D2 (AR)
12	*FANCE*	chr6:35420137-35434881		Fanconi anemia E (AR)
13	*FANCF*	chr11:22644078-22647387		Fanconi anemia F (unknown)
14	*FREM2*	chr13:39261172-39461267	CRL	Fraser syndrome (AR)
15	*GLI3*	chr7:42000547-42276618	VARL	Pallister-Hall syndrome (AD)
16	*IFT172*	chr2:27667239-27712571	ACTERL	Short-rib thoracic dysplasia (AR)
17	*MID1*	chrX:10413349-10851809		Opitz G/BBB syndrome (XLR)
18	*MYCN*	chr2:16080682-16087129		Feingold syndrome (AD)
19	*RFX6*	chr6:117198375-117253326		Mitchell-Riley syndrome
20	*SALL1*	chr16:51169885-51185183	ARL	Townes-Brocks syndrome (AD)
21	*SALL4*	chr20:50400582-50419048	VACRL	Duane-radial ray (AD)
22	*SOX2*	chr3:181429711-181432223	VTEL	Syndromic Microphtalmia (AD)
23	*TBX5*	chr12:114791734-114846247		Holt-Oram syndrome (AD)
24	*WDR35*	chr2:20110028-20189884		Short-rib thoracic dysplasia (AR)
25	*WNT5A*	chr3:55499742-55521670	VARL	Robinow syndrome (AD)
26	*ZIC3*	chrX:136648345-136654259	VCL	Heterotaxy (XLR), VACTERL-H (XLR)
**Candidate genes from the literature in relation to VACTERL, ARM, OR EA/TEF**				
27	*FGF8*	chr10:103529886-103540126	VCRL	Hypogonadotropic hypogonadism (AD)
28	*FOXF1*	chr16:86544132-86548070		Alveolar capillary dysplasia with misalignment of pulmonary veins (AD)
29	*GLI2*	chr2:121554866-121750229		Pallister-Hall syndrome (AD)
30	*PTF1A*	chr10:23481459-23483181		Pancreatic agenesis (AR)
31	*PCSK5*	chr9:78505559-78977255	VACTERL	
32	*RARA*	chr17:38465422-38513895		Leukemia (unknown)
33	*RARB*	chr3:25469753-25639422		Microphthalmia (AD, AR)
34	*SHH*	chr7:155595557-155604967	VACTERL	Holoprosencephaly (AD)
35	*TBX4*	chr17:59533806-59561664		Small patella syndrome (AD)
36	*TRAP1*	chr16:3708037-3767598		
**Genes that cause VACTERL anomalies in mice when mutated**				
37	*ACVR2B*	chr3:38495789-38534633	VCR	Visceral heterotaxy (unknown)
38	*ALDH1A2*	chr15:58245621-58358121	VCRL	
39	*CTNNB1*	chr3:41240941-41281939	VTERL	Mental retardation (AD)
40	*CYP26A1*	chr10:94833231-94837641	VRL	
41	*EFNB2*	chr13:107142078-107187388	VATER	
42	*FOXC1*	chr6:1610680-1614129	VCRL	Axenfeld-Rieger syndrome (AD)
43	*FOXC2*	chr16:86600856-86602537	VCR	Lymphedema-dystichiasis syndrome (AD)
44	*FUZ*	chr19:50310123-50316567	CTEL	Neural tube defects (AD)
45	*HIF1A*	chr14:62162118-62214977	VRL	
46	*HOXD13*	chr2:176957531-176960666	VRL	Syndactyly (AD)
47	*IFT88*	chr13:21141207-21265576	CRL	
48	*ITGAV*	chr2:187454789-187545629	CRL	
49	*KIF3A*	chr5:132028322-132073265	VRL	
50	*NKX2-1*	chr14:36985603-36989430	TERL	Chorea (AD), Choreoathetosis (AD)
51	*NOG*	chr17:54671059-54672951	VATERL	Multiple synostoses syndrome 1 (AD)
52	*PAX3*	chr2:223064605-223163715	VCL	Waardenburg syndrome (AD)
53	*RIPK4*	chr21:43159528-43187249	ATEL	Popliteal pterygium syndrome (AR)
54	*SOX4*	chr6:21593971-21598849	VCTEL	
55	*TBX1*	chr22:19744225-19771112	VCL	Di George syndrome (AD)
56	*WNT3A*	chr1:228194722-228248972	VRL	

*KO, knock-out; V, vertebral; A anal C, cardiac; TE, trachea/esophagus; R, renal; L, limb; ARM, anorectal malformation; EA/TEF, esophageal atresia with or without trachea-esophageal fistula*.

* *The mouse knockout phenotype is only presented if three or more VACTERL component features were affected*.

### Molecular Inversion Probe (MIP)-Based Sequencing

MIPs are 70-nucleotide (nt) single stranded DNA molecules, including a 30-nt common linker sequence and a locus-specific extension and ligation arm of 20-nt each that are complementary to the target DNA. A total of 3000 MIPs were designed to cover the coding regions of the 56 candidate genes. The DNA samples of all patients were analyzed with targeted sequencing using these MIPs, according to previously reported protocols (39–42). MIP capture and the subsequent polymerase chain reaction were performed as described before (39), with minor modifications. The MIP-captured next generation sequencing (NGS) libraries of 419 patients (CURE-Net and Rotterdam) were sequenced on the Next-Seq500 sequencer (Illumina), while 96 patients (AGORA, Cincinnati, and Milan) were sequenced on the HiSeq 2000 sequencer (Illumina). NGS reads were mapped to UCSC human reference genome assembly hg19 and the average coverage of the entire MIP panel was >150-fold per patient. Variants were called and annotated as described previously (41, 42).

### Variant Filtering, Prioritization, and Validation

Variant filtering and prioritization was performed to identify rare variation thought to be damaging for protein function. Positions with <10x coverage were marked as missing due to insufficient coverage. All variants were filtered and prioritized based on the filters presented in Table 2.

**Table 2:** Filtering settings for variant selection with MIP data.

**Filtering settings**	
Quality by Depth	>500
SNP frequency (ExAC and dbSNP 144)	<0.1%
Frequency of in-house exome data (>5000 Dutch exomes)	<0.1%
Gene component	Exon region (only non-synonymous), canonical splice sites
Number of overlaps (sample count in same experiment)	<10
Allele frequency in GoNL database	<0.1%
% Variance (to identify heterozygous variants)	20–80%

*MIP, molecular inversion probes; ExAC, The Exome Aggregation Consortium; dbSNP, database for nucleotide variants; GoNL, Genome of the Netherlands*.

We focused primarily on loss-of-function (LoF) variants, including stop-gains, canonical splice-sites, and frameshifts. We only searched for additional missense variants in genes in which we identified a LoF variant. Subsequently, missense variants were prioritized only when the Combined Annotation Dependent Depletion (CADD) score was >20 and the population frequency was <0.1% in the ExAC and dbSNP databases. Prioritized variants were validated using Sanger sequencing. If available, we included parental DNA in the validation step to determine the segregation of the variant in the family.

### Genotype-Phenotype Correlation and Reverse Phenotyping

When we validated a variant in a gene associated with a monogenic disorder, the patients were carefully re-evaluated in light of the possible new diagnosis based on the genetic information. In case the variant was inherited from one of the parents, the parent's phenotype was also re-evaluated to see whether the segregation in the family would fit the phenotype in the family.

## Results

### Patient Characteristics

In total, 510 patients were analyzed, of whom 211 patients diagnosed with VACTERL, 204 with ARM, and 95 with EA/TEF (Table 3). In the VACTERL cohort, 93 patients were categorized as STRICT-VACTERL patients, 52 as VACTERL-LIKE patients, and 66 as VACTERL-PLUS patients. In the ARM cohort, 119 (58%) patients had additional congenital anomalies, and in the EA/TEF cohort43 (45%) patients. The presence of additional congenital anomalies (VACTERL-PLUS, ARM-PLUS, and EA/TEF-PLUS) is considered a first clue for pediatricians to consider an alternative clinical diagnosis.

**Table 3:** Description of the three different cohorts.

**Cohort**	***N* (%)**
VACTERL	211 (100%)
STRICT-VACTERL	93 (44%)
VACTERL-LIKE	52 (25%)
VACTERL-PLUS	66 (31%)
ARM	204 (100%)
Isolated ARM	85 (42%)
ARM-PLUS*	119 (58%)
EA/TEF	95 (100%)
Isolated EA/TEF	52 (56%)
EA/TEF-PLUS*	43 (45%)
Total	510 (100%)

*ARM, anorectal malformation; EA/TEF, esophageal atresia with or without trachea-esophageal fistula*.

* *ARM and EA/TEF-PLUS: ARM or EA/TEF with additional congenital anomalies*.

### Prioritized Variants

This study revealed five LoF variants in SAL-Like Transcription Factors 1 and 4 (*SALL1, SALL4*) and Midline 1 (*MID1*), genes associated with monogenic disorders with overlapping features (Table 4). In addition, two missense variants with a CADD score >20 and population frequency <0.1% were identified in *SALL4* and *MID1*. These seven variants are considered pathogenic (LoF) or highly pathogenic (missense variants), suggesting an alternative diagnosis in these patients based on genetic evidence. Two of these variants were *de novo* in sporadic cases, four variants were inherited from a mildly affected parent, and one variant had unknown inheritance.

**Table 4:** Loss-of-function and missense variants identified in candidate genes.

**gDNA level**	**cDNA level**	**Protein level**	**Variant**	**Gene**	**CADD score**	**Inheritance**	**Cohort**	**ID**	**Phenotype patient**
Chr16:g.51173039_51173040del	c.3095_3096del	p.(Thr1032 Serfs*11)	Frameshift	*SALL1*	14.1	*De novo*	ARM-PLUS	1	ARM (perineal fistula), bilateral auricular appendage, and right duplication of thumb phalanx
Chr16:g.51175430del	c.703del	p.(Ala235Leufs*12)	Frameshift	*SALL1*	24.3	Maternal	ARM-PLUS	2	ARM (perineal fistula), atrial septal defect, agenesis of the septum pellucidum, right single transverse palmar crease, flat feet, and postnatal cerebral hemorrhage leading to hydrocephalus
Chr16:g.51174083G>A	c.2050C>T	p.(Gln684*)	Stop-gain	*SALL1*	37.0	Unknown	ARM	3	Isolated ARM (perineal fistula)
Chr20:g.50407122T>A	c.1900A>T	p.(Lys634*)	Stop-gain	*SALL4*	40.0	Maternal	ARM	4	Isolated ARM (recto-vestibular fistula)
Chr20:g.50407359C>T	c.1663G>A	p.(Val555Met)	Missense	*SALL4*	22.7	Maternal	ARM-PLUS	5	ARM (vestibular fistula), double left thumb, and an anomaly of the filum terminale
ChrX:g.10427805dup	c.1328dup	p.(Lys444Glnfs*32)	Frameshift	*MID1*	18.5	*De novo*	ARM-PLUS	6	ARM (perineal fistula), tricuspid valve insufficiency, and hypospadia
ChrX:g.10423004G>A	c.1561C>T	p.(Arg521Cys)	Missense	*MID1*	24.0	Maternal	VACTERL-PLUS	7	single umbilical artery, split vertebrae (Th5), ARM, pulmonary vein and artery stenosis, ventricular septal defect, renal dysplasia and left cystic kidney, malrotation, cryptorchidism, mild hypertelorism, and cow's lick.

*Genomic position is based on genome build hg19. ARM, anorectal malformation*.

### SALL1

Variants in *SALL1* are associated with Townes-Brocks syndrome (OMIM #107480), an autosomal dominant disorder characterized by ARM, thumb anomalies, renal anomalies, cardiac anomalies, dysplastic ears, and hearing loss.

A frameshift variant in *SALL1* was identified in an ARM-PLUS patient with a perineal fistula, bilateral auricular appendage, and right duplication of thumb phalanx (ID = 1). The variant was *de novo* and the parents of the patient were healthy. As this family was lost to follow up, clinical re-evaluation was not possible. Based on the “classic” combination of phenotypic features in combination with the genetic evidence, however, this patient can be diagnosed retrospectively with Townes-Brocks syndrome.

A maternally-inherited frameshift variant in *SALL1* was identified in an ARM-PLUS patient with a perineal fistula, atrial septal defect, agenesis of the septum pellucidum, right single transverse palmar crease, flat feet, and postnatal cerebral hemorrhage leading to hydrocephalus (ID = 2). The mother had narrow flat feet, second toes longer than first toes, and chronic kidney failure (IgA nephropathy). During re-evaluation, the sister of this patient was found to be affected with bilateral hearing loss due to adenoid hypertrophy and having an accessory finger without bone on the right ulna site. However, she did not carry the frameshift variant in *SALL1*. Based on this variant and the phenotype, the patient and his mother were diagnosed retrospectively with Townes-Brocks syndrome (43). Due to the highly variable phenotype of Townes-Brocks syndrome, it is not surprising that this patient was not identified based on the phenotype alone.

In a third patient with isolated ARM with a perineal fistula (ID = 3), a stop-gain variant was identified in *SALL1*. Unfortunately, this family was lost to follow up and no parental DNA was available. At the time of recruitment, both the father and sister were noted to have anal atresia. Based on the pathogenic variant in *SALL1* and the fact that ARM is a major clinical feature of Townes-Brocks syndrome (43), the patient was diagnosed with this syndrome. The positive family history in the father and sister makes an autosomal-dominant inheritance likely, although this could not be confirmed.

### SALL4

Variants in the *SALL4* gene are associated with Duane-radial ray syndrome (OMIM #607323), an autosomal dominant disorder characterized by upper limb, ocular, and renal anomalies. Less common features include sensorineural deafness and gastrointestinal anomalies, such as ARM.

A maternally-inherited stop-gain variant was identified in *SALL4* in a patient with isolated ARM with a recto-vestibular fistula (ID = 4). The mother has a left-sided short thumb with clinodactyly of thumb. During clinical re-evaluation, we found that the patient has a younger brother with ARM, meconium plug syndrome, Meckel's diverticulum, ear malformation with hearing loss, accessory thumb, and ventricular septal defect, who was clinically diagnosed with Duane-radial ray syndrome. Subsequently, the brother was found to carry the same *SALL4* variant. As all three family members share a pathogenic variant in *SALL4* and present with features of the Duane-radial ray syndrome (44), this diagnosis seems appropriate. However, it was not suspected for the index patient before evaluating the sequencing data.

A maternally-inherited missense was identified within *SALL4* in an ARM-PLUS patient with vestibular fistula, a double left thumb, and an anomaly of the filum terminale (ID = 5). Besides having bilateral clinodactyly of the fifth finger, the mother of this patient was healthy. The maternal grandmother had clinodactyly as well, but could not be tested. Based on the clinical phenotype and the presence of a missense variant in *SALL4* that is highly likely to be deleterious, a diagnosis of Duane-radial ray syndrome seems appropriate for this patient. The mild phenotype of the mother can be explained by reduced penetrance, as only 13% of patients show the complete triad originally described for Duane-radial ray syndrome (Duane anomaly, radial ray malformation, and sensorineural hearing loss) (44).

### MID1

Variants in the *MID1* gene are associated with the X-linked recessive Opitz G/BBB syndrome (OMIM #300000), in which ARM, cardiac anomalies, TEF, hypospadias, hypertelorism and syndactyly are described.

A frameshift variant in *MID1* was identified in an ARM-PLUS patient with perineal fistula, tricuspid valve insufficiency, and hypospadia (ID = 6). The variant was *de novo* and the parents of the patient were healthy at the time of recruitment. As the family was lost to follow-up, clinical re-evaluation was not possible. It is unknown whether the patient had hypertelorism, a feature present in virtually all affected individuals (45). Based on the mutation identified in *MID1*, however, a diagnosis of Opitz G/BBB syndrome seems most likely for this patient.

A maternally-inherited missense variant was identified in a male VACTERL-PLUS patient with a single umbilical artery, split vertebrae (Th5), ARM, pulmonary vein and artery stenosis, ventricular septal defect, renal dysplasia and left cystic kidney, malrotation, and cryptorchidism (ID = 7). At the time of re-evaluation of the patient's phenotype mild hypertelorism and a cow's lick were noted. Besides having clubfoot and syndactyly of the toes, his mother was healthy and had no hypertelorism, which is consistent with X-linked recessive inheritance. Given the phenotype, the likely damaging genetic variant, and the X-linked recessive segregation in this family, a diagnosis of Opitz G/BBB syndrome seems most likely.

### Variants in the Remaining Candidate Genes

LoF variants were not identified in any of the remaining candidate genes. Therefore, we did not evaluate the presence of missense variants with a CADD score >20 and population frequency <0.1% in these genes.

## Discussion

In this study, the coding regions of 56 candidate genes were sequenced in 510 patients with VACTERL, ARM, or EA/TEF. In the majority of patients, no monogenic disorders were identified that have phenotypic overlap with VACTERL. As we did not find LoF variants in the remaining candidate genes either, we were not able to identify a disease gene for VACTERL based on this gene panel. In seven patients, however, our genetics-first approach provided evidence for a monogenic disorder, allowing the patients to be diagnosed retrospectively with Townes-Brocks, Duane-radial ray, or Opitz G/BBB syndrome.

A strength of this study is that we evaluated a large set of well-characterized VACTERL, ARM, and EA/TEF patients who were clinically diagnosed by pediatric surgeons and/or clinical geneticists. Detailed genetic testing had not been performed in these patients, and they were not suspected of having an alternative disorder. Therefore, it is interesting that we identified several patients affected by an undiagnosed monogenic disorder in this patient population.

The patients in which a monogenic disorder was identified presented either with a milder phenotype or with an atypical presentation of the specific disorders, possibly due to reduced penetrance (46). Therefore, it is not surprising that these patients were not diagnosed correctly before. Our findings suggest that a genetics-first approach seems beneficial in making more accurate diagnoses. Although we identified only seven patients with a monogenic disorder, this information is extremely important for counseling of the families regarding the index patient and the risks in future pregnancies.

At present, VACTERL is considered a diagnosis “*per exclusionem*,” as no genes have been found to confirm the presence of VACTERL based on molecular evidence. This suggests that the diagnosis can only be made when monogenic disorders with multiple features in common with VACTERL are excluded based on the absence of key features and genetic evidence for these monogenic disorders. We hypothesized that a genetics-first approach would improve the identification of patients with monogenic disorders that are hard to distinguish from VACTERL based on the clinical phenotype alone. As we aimed to identify genes that would directly lead to a diagnosis, variant interpretation was rather stringent, with a strong focus on LoF variants. As a result, we were indeed able to identify an alternative diagnosis based on genetic evidence in 7 out of 510 patients (1.4%). In 5 out of these 7 patients, additional congenital anomalies were present, confirming our hypothesis that the presence of additional congenital anomalies is a first clue for pediatricians to consider an alternative diagnosis.

This study was not population-based, however, so the frequency of monogenic disorders in all patients with features of the VATER/VACTERL spectrum may be different. In addition, it is worth mentioning that most of the patients in which we identified an alternative diagnosis came from the ARM cohort (6/204 = 2.9%). As the clinical phenotype of ARM is quite variable, this may be the best cohort to use in a genetics-first approach.

We did not identify any LoF variants in the remaining candidate genes, but our gene panel covered only a small proportion of the human genome. The use of genome-wide approaches, such as WES or WGS, may be more fruitful in identifying new genes or genetic regions that are associated with VACTERL or its individual features ARM or EA/TEF. The beneficial effects of a genotype-first approach have been demonstrated for other sporadic heterogeneous disorders, such as intellectual disability, autism, and schizophrenia, as well (47, 48). Trio analyses may highlight the role of *de novo* variation, as most of the VACTERL cases occur sporadically.

Ten of the candidate genes from our gene panel were previously reported in the literature and were used to screen groups of VACTERL patients before. Based on a promising knock-out animal model and variants identified in VACTERL patients, the Proprotein Convertase Subtilisin/Kexin Type 5 gene (*PCSK5*) was considered a very interesting candidate (27, 37). However, we did not identify LoF variants in *PCSK5*, which is in line with a previous study among 39 VACTERL patients (15). Variants in other candidate genes, including Sonic Hedgehog (*SHH*), Pancreas Associated Transcription Factor 1a (*PTF1A*), and LIM Domain Containing Preferred Translocation Partner in Lipoma (*LPP*) were not found either, in agreement with the literature (12, 14, 35). In most studies, including this one, only the coding regions of the candidate genes were sequenced. It is possible, however, that non-coding regions of these genes play a role in the etiology of complex congenital anomalies, such as VACTERL. As non-coding regions often contain regulatory elements involved in gene expression, they might be of interest to study. In addition, epigenetic factors, such as DNA methylation, may also play an important role.

In conclusion, we did not find an unequivocal disease gene for VACTERL, ARM, or EA/TEF. The lack of identifying mutations in one or a few genes in a large percentage of patients confirms that the VACTERL association remains a difficult to tackle heterogeneous group of rare disorders. However, we did identify seven patients with monogenic disorders that were clinically not recognized as such, using a genetics-first approach. Although the proportion of patients that received an alternative diagnosis through genetic testing was low, we would still like to highlight the importance of genetic testing in patients with multiple congenital anomalies to prevent misdiagnosis, as this is extremely important for counseling of the families. This study exemplifies that a phenotypically rather broad cohort benefits from a genotype-first approach to define the molecular diagnosis in a subset of cases.

## Data Availability Statement

The raw data supporting the conclusions of this article can be made available by the authors upon individual request, without undue reservation.

## Ethics Statement

Written informed consent was obtained from all participants and/or their parents. The Ethics Committees of the University of Bonn and the University of Heidelberg approved the CURE-Net study protocol, the Regional Committee on Research Involving Human Subjects Arnhem-Nijmegen approved the AGORA study protocol, the Erasmus University Medical Centre's local ethics board approved the Erasmus study protocol, and the Institutional Review Board at the Cincinnati Children's Hospital Medical Center approved the collection of genetic samples of the Cincinnati patients. Written informed consent to participate in this study was provided by the participants' legal guardian/next of kin.

## Author Contributions

RP carried out the final analyses, interpreted the data and drafted the initial manuscript. GD was involved in the acquisition of data by performing clinical re-evaluations of some of the patients and interpreted the data. EB, AK, HR, and CM conceptualized and designed the study. NR, IR, HB, and AH conceptualized and designed the study, interpreted the data, and had general supervision of the project. RA conceptualized and designed the study and carried out the initial analyses. NK carried out the initial experiments and analyses. MS carried out validation experiments. SD was involved in conceptualization of the study. ES, SM, NS, CS, AB, DT, GB, AM, MFB, MDB, ML, AP, and IB were involved in the acquisition of data by including research participants. All authors revised the manuscript for important intellectual content, approved the final manuscript as submitted, and agreed to be accountable for all aspects of the work.

## Conflict of Interest

The authors declare that the research was conducted in the absence of any commercial or financial relationships that could be construed as a potential conflict of interest.

## Abbreviations

ARM: Anorectal malformation
AGORA: Aetiologic research into Genetic and Occupational/environmental Risk factors for Anomalies in children
CADD: Combined Annotation Dependent Depletion
CURE-Net: The German Network for Congenital Uro-REctal malformations
EA: Esophageal atresia
LoF: Loss-of-Function
MID1: Midline 1
MIPs: Molecular inversion probes
SALL1: SAL-Like Transcription Factors 1
SALL4: SAL-Like Transcription Factors 4
TEF: Trachea-esophageal fistula
WES: Whole exome sequencing
WGS: Whole genome sequencing.
